# Hepatic resection for recurrent hepatocellular carcinoma during pregnancy: a case report

**DOI:** 10.1186/s40792-020-00985-9

**Published:** 2020-09-29

**Authors:** Takashi Maeda, Daisuke Imai, Huanlin Wang, Kyohei Yugawa, Nao Kinjo, Koto Kawata, Shinichiro Ikeda, Keitaro Edahiro, Kazuki Takeishi, Tomohiro Iguchi, Hiroto Kayashima, Noboru Harada, Mizuki Ninomiya, Shohei Yamaguchi, Kozo Konishi, Shinichi Tsutsui, Hiroyuki Matsuda

**Affiliations:** 1 Department of Surgery, Hiroshima Red Cross Hospital and Atomic-Bomb Survivors Hospital, 1-9-6 Senda-machi, Naka-ku, Hiroshima, 730-8619 Japan; 2 Department of Surgery and Science, Graduate School of Medical Sciences, Kyushu University, 3-1-1 Maidashi, Higashi-ku, Fukuoka, 810-8582 Japan

**Keywords:** Hepatocellular carcinoma, Pregnancy, Hepatic resection

## Abstract

**Background:**

Hepatocellular carcinoma (HCC) during pregnancy is extremely rare. Treatment strategies for cancers detected during pregnancy have been controversial. We herein report a case of recurrent HCC detected at 20 weeks of pregnancy, which subsequently prompted hepatic resection after abortion.

**Case presentation:**

A 36-year-old woman underwent laparoscopic partial hepatectomy for HCC (20 mm in diameter) in segment 5 of the liver during follow-up after being determined as a hepatitis B virus carrier two and a half years ago. Post-surgery follow-up abdominal ultrasonography revealed a 36-mm tumor in segment 7 of the liver. Abdominal contrast-enhanced computed tomography revealed a well-enhanced tumor with a 40-mm diameter in segment 7 adjacent to the inferior vena cava and right hepatic vein, suggesting HCC recurrence. Laboratory data revealed total bilirubin (0.4 mg/dL), aspartate aminotransferase (28 IU/L), alanine aminotransferase (30 IU/L), glutamyltransferase (16 IU/L), prothrombin time (115.3%), and indocyanine green retention rate at 15 min (7.0%). α-Fetoprotein (AFP) (12,371.5 ng/mL; normal range < 10 ng/mL) and PIVKA-II (208 mAU/mL; normal range < 40 mAU/mL) were both significantly elevated. After discussions with a cancer board consisting of experts from the departments of gastroenterology, obstetrics and gynecology, and surgery, as well as obtaining appropriate informed consent from the patient and her family, we decided to perform a hepatic resection after abortion. Subsequently, abortion surgery was performed at 21 weeks and 2 days of pregnancy. After 6 days, subsegmentectomy of liver segment 7 was performed under general and epidural anesthesia, with a pathological diagnosis which was moderately differentiated HCC being established. Given the good postoperative course, without particular complications, the patient was subsequently discharged 10 days after the operation. Approximately 2 years after the surgery, the patient remains alive without recurrence, while both AFP and PIVKA-II were within normal limits.

**Conclusions:**

Treatment strategies for HCC detected during pregnancy remain controversial. As such, decisions should be made based on HCC growth and fetal maturity after thorough multidisciplinary team discussions and obtaining appropriate informed consent from the patient and her family.

## Background

While extremely rare, hepatocellular carcinoma (HCC) during pregnancy has a poor prognosis. Treatment strategies for cancers detected during pregnancy remain controversial. We herein report a case of recurrent HCC detected at 20 weeks of pregnancy, which subsequently prompted hepatic resection after abortion.

## Case presentation

**Fig. 1:**
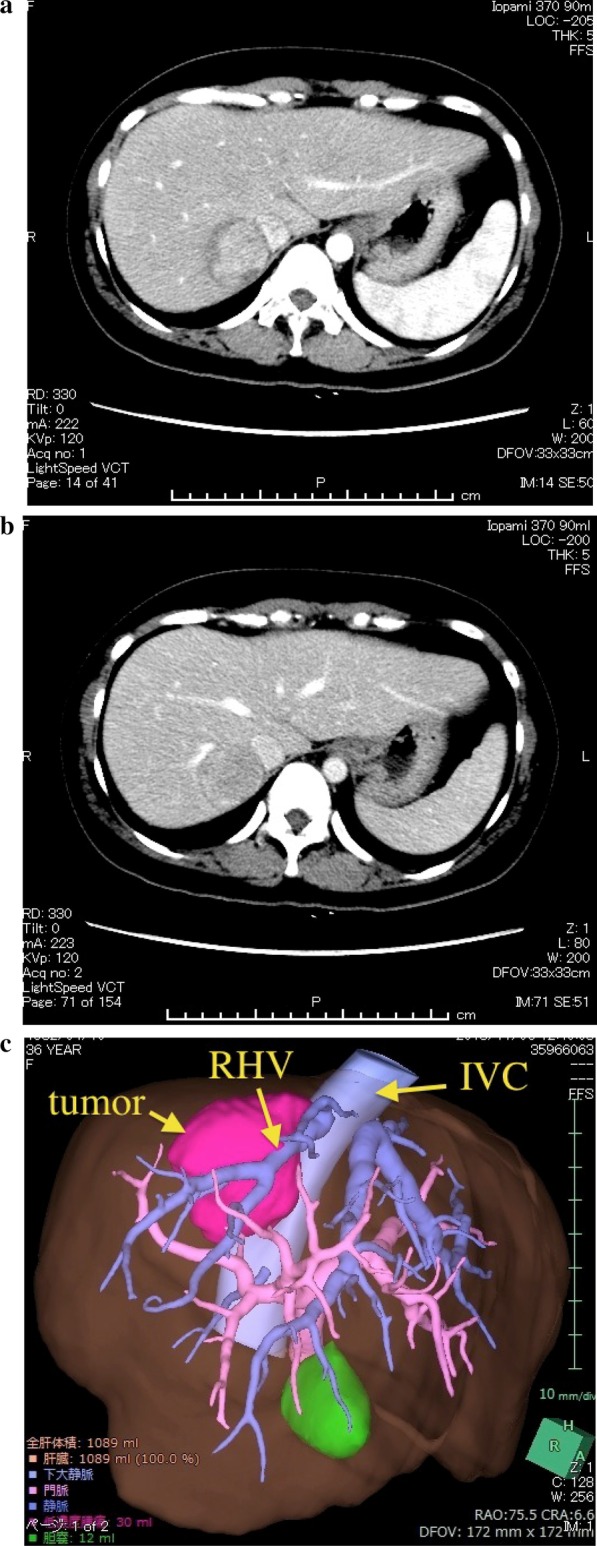
Abdominal contrast-enhanced computed tomography revealing a 40-mm tumor, which was **a** well enhanced in the arterial phase and **b** washed out in the portal venous and delayed phases, suggesting hepatocellular carcinoma recurrence. The three-dimensional image (VINCENT) showed the tumor adjacent to the inferior vena cava and right hepatic vein (**c**)

**Fig. 2:**
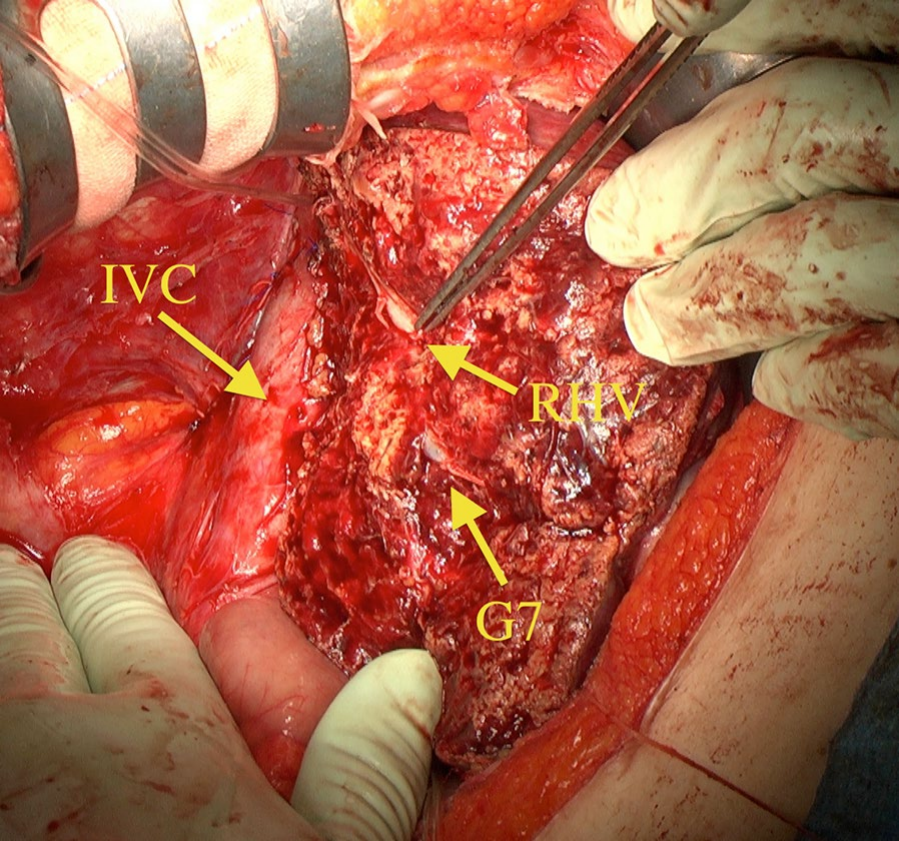
We continued hepatic resection along right hepatic vein (RHV), exfoliated a tumor within the vicinity of a root of RHV, and, subsequently, completed subsegmentectomy of liver segment 7

**Fig. 3:**
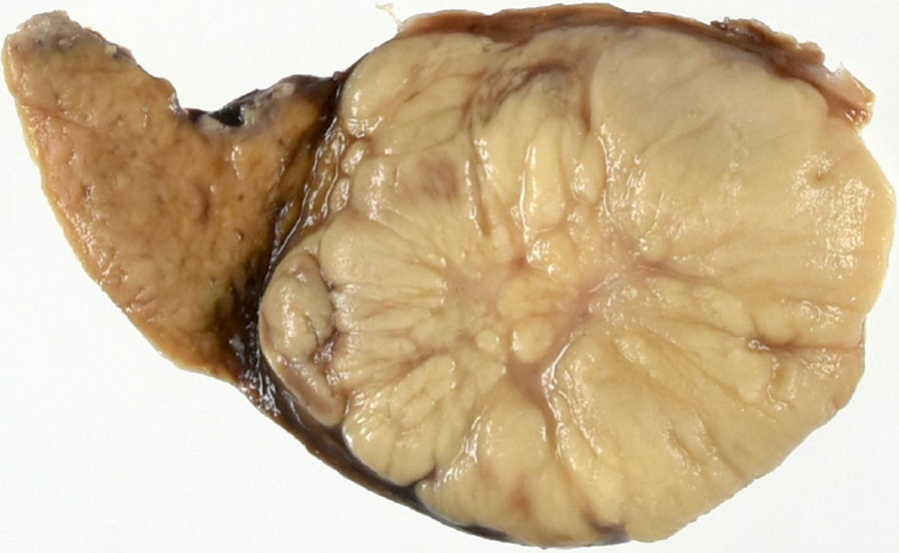
Macroscopic findings of the tumor. The whitish tumor with a maximum diameter of 4.0 cm was simple nodular type and showed expansive growth

A 36-year-old woman underwent a laparoscopic partial hepatectomy for HCC (20 mm in diameter) in liver segment 5 two and a half years ago during follow-up after having been identified as a hepatitis B virus carrier. She has been treated for hepatitis B with nucleoside analogue before the hepatectomy. A pathological diagnosis of moderately differentiated HCC was subsequently established. We followed up at interval of 2–3 months after the hepatectomy. During post-surgery follow-up, abdominal ultrasonography detected a 36-mm hypoechoic tumor in segment 7 of the liver. Abdominal contrast-enhanced computed tomography revealed a 40-mm tumor adjacent to the inferior vena cava (IVC) and right hepatic vein (RHV). The tumor was well enhanced in the arterial phase and washed out in the portal venous and delayed phases, suggesting HCC recurrence (Fig. 1). No nodule suggesting HCC was identified elsewhere in the liver. Neither lymph-node swelling nor metastasis to other organs was detected. The following laboratory data were obtained: white blood cell count (3000/µL), hemoglobin (10.1 g/dL), platelet count (16.5 × 10^4^/µL), C-reactive protein (2.98 mg/dL), urea nitrogen (9.2 mg/dL), creatinine (0.51 mg/dL), total protein (6.3 g/dL), albumin (3.1 g/dL), total bilirubin (0.4 mg/dL), aspartate aminotransferase (28 IU/L), alanine aminotransferase (30 IU/L), glutamyltransferase (16 IU/L), lactate dehydrogenase (179 U/L), alkaliphosphatase (192 U/L), prothrombin time (115.3%), and indocyanine green retention rate at 15 min (7.0%). α-Fetoprotein (AFP) (12,371.5 ng/mL; normal range < 10 ng/mL) and PIVKA-II (208 mAU/mL; normal range; < 40 mAU/mL) were both significantly elevated. Considering that the patient was still 20 weeks pregnant, delivery was not viable. However, surgery could not be delayed any longer given the rapid tumor growth, which could likely be associated with pregnancy. After discussion with at a cancer board consisting of experts from the departments of gastroenterology, obstetrics and gynecology, and surgery, as well as obtaining appropriate informed consent from the patient and her family, we decided to perform hepatic resection after abortion. Subsequently, abortion surgery was performed at 21 weeks and 2 days of pregnancy, followed by subsegmentectomy of liver segment 7 under general and epidural anesthesia 6 days later. Mental care for the patient was provided by obstetricians, surgeons, and nurses before and after abortion. After mobilizing the right lobe of the liver, we identified the Glissonian pedicle (G) 7 and injected indigo carmine under US guidance to mark the perfusion area. Thereafter, hepatic resection was initiated using an ultrasonic dissector under the Pringle’s maneuver. After identifying the G7, a clamp test was performed to reconfirming the ischemic area before ligating and cutting it. We continued hepatic resection along the RHV, exfoliated a tumor within the vicinity of a root of the RHV, and subsequently completed subsegmentectomy of liver segment 7 (Fig. 2). The surgery lasted 274 min, with an estimated blood loss of 940 mL. Macroscopically, the whitish tumor with a maximum diameter of 4.0 cm was simple nodular type and showed expansive growth (Fig. 3). A pathological diagnosis of moderately differentiated HCC, grade II, eg, fc (−), sf (+), s0, vp0, vv0, b0, im0, and sm (−) was thereafter established. Tumor cells showed a moderate degree of atypia, as well as a cord-like or pseudoglandular arrangement associated with abundant eosinophilic vesicles and a rich nucleoplasm. Given the good postoperative course, without particular complications, the patient was discharged 10 days after surgery. Approximately 2 years after surgery, the patient remains alive without recurrence, while both AFP and PIVKA-II were within normal limits.

## Discussion

HCC during pregnancy, which is believed to have a worse prognosis than that among non-pregnant woman, is extremely rare, with only 62 cases having been reported worldwide to date [1–14]. Accordingly, studies have shown an overall 6-month and 1-, 2-, and 3-year survival rates of 50%, 29.5%, 18.2%, and 13.6%, respectively, with a median survival of 18 months in pregnant patients with HCC [2]. Moreover, the previous reports have revealed that 20% of the pregnant patients with HCC developed distant metastasis upon presentation [3]. Although hepatic resection has been the best potentially curative therapy for HCC [1–5], Choi et al. [2] reported that only 16 of 48 pregnant mothers with HCC underwent liver resection. Currently, repeat hepatectomy seems to be a better choice for recurrent HCC. However, no long-term survival after HCC recurrence during pregnancy has been reported in the literature [5]. Nonetheless, the present case survived without recurrence for approximately 2 years after the second hepatectomy for recurrent HCC and 5 years after the first hepatectomy.

Several studies have shown that pregnancy clearly has an adverse effect on the prognosis of HCC [3, 6–8], with only 3 of 29 patients surviving 12 months or more after diagnosis [6, 9]. HCC becomes aggressive during pregnancy, primarily due to two main etiologies: (1) estrogen elevation, which accelerates HCC evolution, and (2) immune suppression during pregnancy [3]. Accordingly, estrogen has been shown to increase hepatocyte mitosis, hypervascularity, and free radicals; reactivate the hepatitis B virus; and decrease humoral immunity [3]. During pregnancy, large amounts of human chorionic gonadotropin, estrogen, and placental lactogen secreted from the placenta are believed to promote the growth and reproduction of cancer cells, thus aggravating the aggressiveness of the underlying HCC [4]. Moreover, gestational immune suppression may be an enabling factor for tumor progression [6, 8].

Previous studies have reported that pregnancy itself could promote rapid HCC growth and increase the risk of tumor rupture [6] and hemorrhage—both of which are life-threatening complications. Indeed, approximately 10% of HCCs during pregnancies are complicated by tumor rupture, perhaps, partly due to tumor liquefaction necrosis, which, together with the increased intra-abdominal pressure secondary to the pregnancy, predisposes patients to rupture and hemorrhage [4, 6–8]. Therefore, delaying treatment until delivery after 32 weeks of gestation could threaten both maternal and fetal survival [1]. Despite our patient being 20 weeks pregnant, we determined that surgery could not be delayed any longer given that the tumor was protruding from the liver surface and had a high risk of rupture.

Treatment strategies for malignant neoplasms detected during pregnancy remain controversial given the need to consider both the mother and the fetus. Accordingly, majority of the reported cases had resulted in termination of pregnancy upon diagnosis of cancer [4, 7, 9, 10]. For pregnancies in the first trimester, maternal survival usually takes precedence. Delaying resection until the 28th week would be futile considering that neither the mother nor the baby will have survived [4, 6, 11]. Therefore, therapeutic abortion should be offered, so that the mother can received treatment as soon as possible [7]. For patients in the second or third trimesters, a thorough discussion with the patient on the management plan should be adopted [7]. Thorough discussions with a multidisciplinary team of surgeons, oncologists, and obstetricians would be necessary to decide the appropriate timing of surgery while considering the potential viability of the fetus. For smaller neoplasms, a delay in resection to allow more fetal maturity may be a reasonable approach [4]. During the third trimester, surgery should not be delayed given that the fetus can be delivered through Cesarean section simultaneously with hepatectomy [4]. The 28th gestational week is the critical point of fetal maturation. Premature birth has been defined as that before the 28th week of gestation, before which survival of the baby is much less probable. Therefore, the gestational age of the baby plays a major role in decision-making [4]. In addition, delivery before 32 weeks of gestation should be avoided because of fetus immaturity [1].

Recently, various surgeries, including hepatectomy, have been safely performed during the second and third trimesters, suggesting that patients do not always need to terminate their pregnancy [1]. We think that the important points to note in hepatectomy of pregnant women when performing hepatectomy while continuing pregnancy are to avoid pressure on the uterus, shorten the operation time, and reduce blood loss. Preoperative simulation of hepatectomy and monitoring fetal heartbeat are essential. Complications of anesthesia should also be considered. In this case, after discussions with a cancer board consisting of experts from the departments of gastroenterology, obstetrics and gynecology, and surgery, as well as obtaining appropriate informed consent from the patient and her family, we decided to perform a hepatic resection after therapeutic abortion. Considering that the patient was still 20 week pregnant, delivery was not viable. However, surgery could not be delayed any longer given the rapid tumor growth, such that rupture was imminent, which was likely associated with pregnancy. Moreover, given that the tumor was in contact with the IVC and RHV, massive bleeding during surgery could cause a sudden drop in blood pressure, endangering both maternal and fetal life. Despite other opinions regarding the treatment choice, hepatic resection had been performed after abortion, prioritizing the rapidly growing HCC and maternal safety.

## Conclusions

HCC during pregnancy is extremely rare. Moreover, pregnancy itself could promote rapid HCC growth and increase the risk of tumor rupture, leading to poor prognosis. Given the controversial treatment strategies for HCC detected during pregnancy, decisions should be made based on HCC growth and fetal maturity after thorough multidisciplinary team discussions and obtaining appropriate informed consent from the patient and her family.

## Data Availability

All datasets supporting the conclusions of this article are included in this published article.

## Abbreviations

HCC: Hepatocellular carcinoma
AFP: α-Fetoprotein
IVC: Inferior vena cava
RHV: Right hepatic vein
G: Glisson pedicle
